# Neoadjuvant fractionated stereotactic radiotherapy followed by piecemeal resection of brain metastasis: a case series of 20 patients

**DOI:** 10.1007/s10147-021-02083-8

**Published:** 2021-11-18

**Authors:** Shoichi Deguchi, Koichi Mitsuya, Kazuaki Yasui, Keisuke Kimura, Tsuyoshi Onoe, Hirofumi Ogawa, Hirofumi Asakura, Hideyuki Harada, Nakamasa Hayashi

**Affiliations:** 1 Division of Neurosurgery, Shizuoka Cancer Center, 1007, Shimo-nagakubo, Naga-izumi, Shizuoka, 411-8777 Japan; 2 Radiation and Proton Therapy Center, Shizuoka Cancer Center, Shizuoka, Japan

**Keywords:** Brain metastasis, Leptomeningeal disease, Neoadjuvant fractionated stereotactic radiotherapy, Piecemeal resection, Radiation necrosis

## Abstract

**Background:**

The safety and effectiveness of neoadjuvant fractionated stereotactic radiotherapy (FSRT) before piecemeal resection of brain metastasis (BM) remains unknown.

**Methods:**

We retrospectively reviewed 20 consecutive patients with BM who underwent neoadjuvant FSRT followed by piecemeal resection between July 2019 and March 2021. The prescribed dose regimens were as follows: 30 Gy (*n* = 11) or 35 Gy (*n* = 9) in five fractions.

**Results:**

The mean follow-up duration was 7.8 months (range 2.2–22.3). The median age was 67 years (range 51–79). Fourteen patients were male. All patients were symptomatic. All tumors were located in the supratentorial compartment. The median maximum diameter and volume were 3.7 cm (range 2.6–4.9) and 17.6 cm^3^ (range 5.6–49.7), respectively. The median time from the end of FSRT to resection was 4 days (range 1–7). Nausea (CTCAE Grade 2) occurred in one patient and simple partial seizures (Grade 2) in two patients during radiation therapy. Gross total removal was performed in seventeen patients and sub-total removal in three patients. Postoperative complications were deterioration of paresis in two patients. Local recurrence was found in one patient (5.0%) who underwent sub-total resection at 2 months after craniotomy. Distant recurrence was found in six patients (30.0%) at a median of 6.9 months. Leptomeningeal disease recurrence was found in one patient (5.0%) at 3 months. No radiation necrosis developed.

**Conclusions:**

Neoadjuvant FSRT appears to be a safe and effective approach for patients with BM requiring piecemeal resection. A multi-institutional prospective trial is needed.

## Introduction

Surgical resection plays a significant role in the management of brain metastasis (BM) for the relief of symptoms and improvement of overall survival (OS) [1–3]. Because surgery alone, with no adjuvant radiotherapy, was insufficient to provide durable local control, postoperative radiation therapies have been recommended. Initially, postoperative whole-brain radiotherapy has been demonstrated to reduce the risk of local recurrence [4]. However, since the reporting of the JCOG0504 study in 2018, postoperative salvage single-fraction stereotactic radiosurgery (SRS) has become the standard treatment of patients with BMs requiring surgery because of its efficacy, convenience, and low toxicity [5]. In clinical practice, postoperative SRS on the margin of the resection cavity is not performed in cases of en bloc tumor resection, but is often performed early in cases of piecemeal resection. Postoperative SRS has been reported to be associated with symptomatic radiation necrosis (RN) rates of 6–26% [6] and leptomeningeal disease (LMD) rates of 11–28% at 1 year [7–11]. In cases of piecemeal resection, there is a need for a new method of radiation therapy for reducing LMD and RN while maintaining adequate local control.

The diagnosis of LMD is associated with a poor prognosis. The surgical strategy has been cited as a risk factor for the development of LMD. Piecemeal resection confers a higher risk of LMD than en bloc resection [12], so en bloc tumor resection should be attempted whenever possible. In clinical practice, however, the choice of piecemeal resection is often unavoidable when the tumor is large in size and/or is close to the eloquent areas. Even in such cases, neoadjuvant stereotactic irradiation may reduce the intraoperative seeding of viable tumor cells beyond the operative field, resulting in a reduced risk of LMD [13, 14].

Symptomatic RN impairs patients’ activities of daily living and quality of life, which may require additional surgical and/or medical treatment [15]. The risk factors of radiation necrosis include radiation dose, the size of the lesion, and the fraction size [16]. Recently, fractionated stereotactic radiotherapy (FSRT) has been reported to reduce the rate of necrosis compared with single-fraction SRS for large resected BMs [10, 17].

To the best of our knowledge, no reports on preoperative FSRT have yet been published in English. In this preliminary study, we retrospectively examined the effectiveness and safety of neoadjuvant FSRT before the piecemeal resection of BM.

## Patients and methods

This study was single-institution retrospective analysis of 20 consecutive patients with BM who underwent neoadjuvant FSRT followed by piecemeal resection between July 2019 and March 2021. The indication for neoadjuvant FSRT was approved by a conference with neurosurgeons and radiation oncologists. The index lesion should be > 2.5 cm in largest diameter. The number of BM should be 1–4 on magnetic resonance imaging (MRI). The treating neurosurgeons determined the need for piecemeal resection based on tumor location, tumor size, and associated symptoms. Patients who required immediate or urgent surgical resection or who could safely undergo en bloc resection were excluded.

The following demographic/clinical data were obtained: date of birth, sex, date of FSRT and craniotomy, primary diagnosis, side/location of BM, symptoms due to BM, resection methods, extent of resection, pre- and postoperative Eastern Cooperative Oncology Group Performance Status (ECOG PS), volume of BM, maximum diameter of BM, prescribed FSRT dose, number of treated lesions, any complication of craniotomy and radiotherapy, local recurrence (LR), distant recurrence (DR), leptomeningeal disease recurrence, radiation necrosis, date of death or last follow-up visit, and cause of death. OS was calculated from the date of craniotomy to death from any cause or the last day of follow-up using Kaplan–Meier methods. Gross total removal was defined as the absence of residual tumor in postoperative MRI and subtotal removal as the presence of ~ 10% residual tumor.

Regarding imaging, the patients were followed using contrast-enhanced MRI at least every 2 months. LR was defined as the presence of new, progressive nodular enhancement within the resection cavity on contrast-enhanced MRI. DR was defined as the presence of new enhanced lesions ≥ 5 mm away from the treated prescription isodose line. LMD was defined as the presence of new leptomeningeal enhancement ≥ 5 mm away from the treated prescription isodose line [13]. RN was defined as three radiological criteria: (1) increased T1 enhancement within the radiation treatment fields, (2) absence of increased vascular flow on perfusion computed tomography (CT), and (3) a decrease of enhancement on follow-up imaging. All MRI studies were reviewed by two neurosurgeons (S.D. and K.M.). Adverse events were graded based on Common Terminology for Adverse Events (CTCAE) version 5.

FSRT was delivered with dynamic conformal arcs with True beam STx (Varian Medical System). The clinical target volume was a zero-margin expansion of the gross total volume. A 1.5-mm-margin volumetric expansion was added to the clinical target volume to generate the planning target volume (PTV). All plans were optimized so that 95% of the PTV received the prescription dose. The amount of all normal tissue outside the PTV receiving more than 20 Gy (V20) was used as an index to determine the prescription dose for BMs [18]. Therefore, we prescribed 30 Gy in five fractions (*n* = 11) for patients with V20 ≥ 23 cc and 35 Gy in five fractions (*n* = 9) for patients with V20 < 23 cc [18]. Statistical analyzes were performed using EZR statistical software [19]. LR free survival, DR free survival, LMD free survival and OS were calculated using the Kaplan–Meier method.

All of these analyses were approved in advance by the institutional review board (IRB; approval number: J2019-194-2019-1-3).

## Results

The characteristics of 20 patients with BM who underwent neoadjuvant FSRT followed by piecemeal resection are summarized in Table 1. The mean follow-up duration was 7.8 months (range 2.2–22.3). The median age at the time of craniotomy was 67 years (range 51–79). Fourteen patients were male. The most common malignancy was non-small-cell lung cancer (9 of 20), followed by esophageal cancer (3 of 20). All patients were symptomatic. All tumors were located in the supratentorial compartment (left frontal: 6, left parietal: 2, right frontal: 5, right parietal: 3, right occipital: 2, right temporal: 1, right lateral ventricle: 1). The median maximum diameter was 3.7 cm (range 2.6–4.9). The median tumor volume was 17.6 cm^3^ (range 5.6–49.7). The median number of lesions treated by FSRT was 2 (range 1–4).

**Table 1:** Patients demographics, tumor characteristics, and outcomes

Age (median)	67 years old (51‒79)
Sex	M: 14, F: 6
Primary diagnosis	NSCLC: 9, SCLC: 1, Esophagus: 3, Colon: 2, Kidney: 2, HCC: 1, Uterus: 1, Melanoma: 1
Tumor location	Lt. frontal: 6, Lt. parietal: 2, Rt. frontal: 5, Rt. parietal: 3, Rt. occipital: 2, Rt. temporal:1, Rt. lateral ventricle: 1
Maximum diameter (median)	3.7 cm (2.6‒4.9)
Tumor volume (median)	17.6 cm3 (5.6‒49.7)
Overall treatment period of radiotherapy (median)	7 days (5‒8)
Time from SRT to Op (median)	4 days (1‒7)
Prescribed dose (median)	30 Gy (30‒35)
Number of treated lesions	2 (1‒4)
EOR	GTR: 17, STR: 3
Preop PS (median)	2 (1‒3)
Postop PS (median)	1 (0‒3)
LR	1/20
DR	6/20
RN	0/20
LMD	1/20
Follow-up (mean)	7.8 months (2.2–22.3)
Outcome	Alive: 12, dead: 8

*SRT* stereotactic radiotherapy, *Op* operation, *PS* performance status, *EOR* extent of resection, *LR* local recurrence, *DR* distant recurrence, *RN* radiation necrosis, *LMD* leptomeningeal disease, *M* male, *F* female, *SCLC* small-cell lung cancer, *HCC* hepatocellular carcinoma, *NSCLC* non-small-cell lung cancer, *Rt.* right, *Lt.* left, *GTR* gross total removal, *STR* subtotal removal

The median FSRT dose was 30 Gy (range 30–35). The median overall treatment period of radiotherapy was 7 days (range 5–8). The median time from the end of FSRT to resection was 4 days (range 1–7). Regarding adverse events, nausea (CTCAE Grade 2) occurred in one patients and simple partial seizures (CTCAE Grade 2) in two patients during radiation therapy. Gross total removal was performed in seventeen patients and sub-total resection in three patients with lesions involving eloquent area. Postoperative complications were deterioration of paresis in two patients. Wound healing failure and cerebrospinal fluid leakage were not occurred. The postoperative mortality rate within 30 days was 0%.

**Fig. 1:**
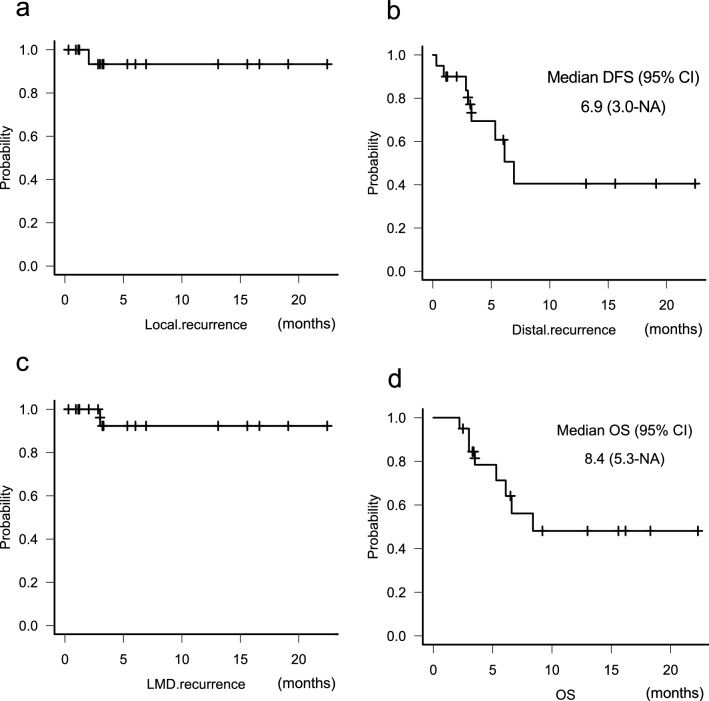
Kaplan–Meier analysis. Kaplan–Meier curve for local recurrence free survival (**a**), distant recurrence free survival (DFS) (**b**), leptomeningeal recurrence (LMD) free survival (**c**) and overall survival (OS) (**d**)

LR was found in one patient (5.0%) who underwent sub-total resection at 2 months after craniotomy (Fig. 1a). DR was found in six patients (30%) at a median of 6.9 months after craniotomy (Fig. 1b). Leptomeningeal disease recurrence was in one patient (5.0%) at 3 months after craniotomy (Fig. 1c). No RN developed in our cohorts. Median OS was 8.4 months (Fig. 1d). Eight patients (40%) died during this study. Six died of aggravation of primary tumor and two died of BM.

### Illustrative case 1

**Fig. 2:**
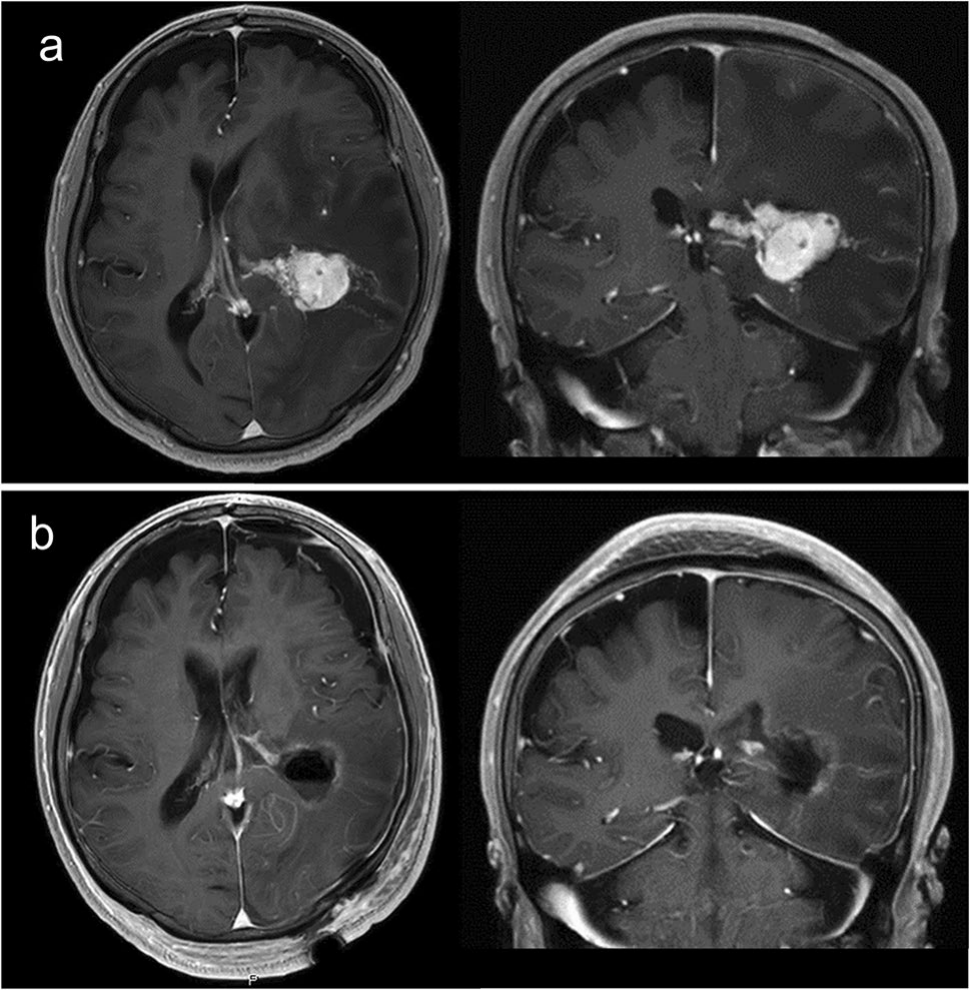
Pre- and postoperative MRI of illustrative case 1. **a** Preoperative axial and coronal T1-weighted MRI with contrast. **b** Postoperative axial and coronal T1-weighted MRI with contrast

A 63-year-old man with renal cell carcinoma visited our hospital due to mild cognitive dysfunction. Head MRI showed a tumor in his left lateral ventricle (Fig. 2a). Our preoperative diagnosis was brain metastasis from renal cell carcinoma. Neoadjuvant FSRT (35 Gy in five fractions) followed by piecemeal resection was performed. The histopathological diagnosis was brain metastasis from renal cell carcinoma. Postoperative MRI showed complete resection of the tumor (Fig. 2b). No evidence of LR or LMD has emerged during more than 22 months.

### Illustrative case 2

**Fig. 3:**
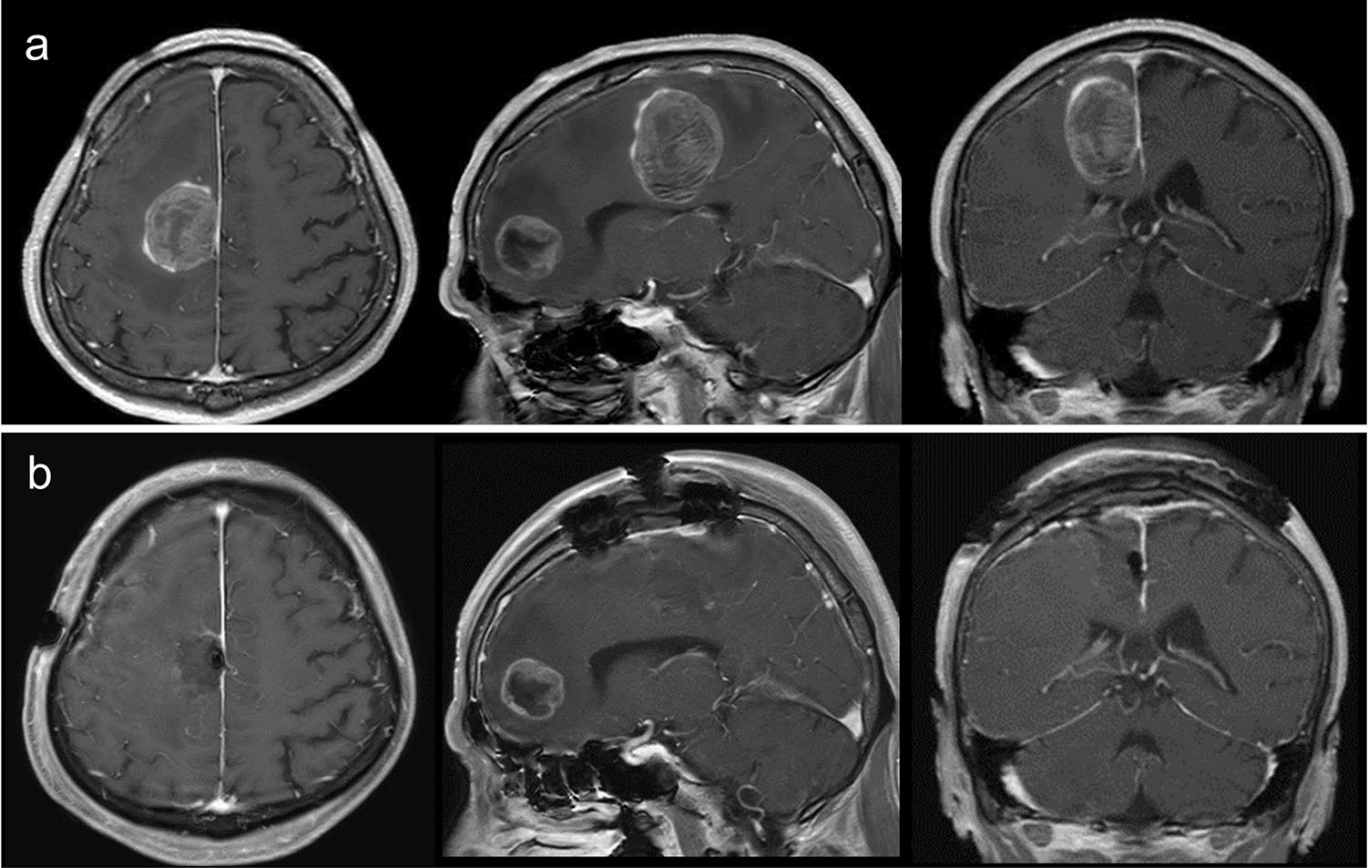
Pre- and postoperative MRI of illustrative case 2. **a** Preoperative axial, sagittal, and coronal T1-weighted MRI with contrast. **b** Postoperative axial, sagittal, and coronal T1-weighted MRI with contrast

A 51-year-old man visited our hospital due to severe left hemiparesis. Head MRI showed two brain tumors in his right frontal lobe. One of them was located in the precentral gyrus (Fig. 3A), the maximum diameter of which was 45 mm. Chest CT also showed a tumor in his left lung. Neoadjuvant FSRT (30 Gy in five fractions) followed by piecemeal resection was performed. Postoperative MRI showed gross total removal of the tumor (Fig. 3B). His left hemiparesis gradually improved. No evidence of LR or LMD has emerged during more than 6 months.

## Discussion

Postoperative salvage SRS is becoming the standard treatment for patients with one to four BMs who require tumor removal, according to the results of the JCOG0504 study [5]. In that study, 35% of subjects in the SRS arm did not receive any postoperative radiation therapy. In clinical practice, postoperative SRS on the margin of the resection cavity is not performed in cases of en bloc tumor resection, but is often performed early in cases of piecemeal resection. The choice of piecemeal resection is often unavoidable when the tumor is large and/or close to the eloquent areas. Radiotherapy combined with surgery may need to be selected depending on the method of tumor removal. Therefore, in this study we included only patients with one to four BMs who underwent resection in a piecemeal manner.

Preoperative FSRT is a potential method of decreasing symptomatic RN while maintaining good local control. FSRT delivers a higher dose of radiation with potentially similar or lower rates of toxicity compared with single-fraction SRS, since fractionation takes radiobiological advantage of normal brain tissue repair and reoxygenation [3]. Minniti et al. analyzed the clinical outcome of 289 patients with large (> 2.0 cm) BM who received SRS or FSRT, with the results revealing that FSRT showed a better 1-year cumulative local control rate (91% versus 77%, *P* = 0.01) and a reduced risk of RN (8% versus 20%, *P* = 0.004) [20]. On the other hand, it has been reported that stereotactic radiotherapy alone deteriorates local control rate as the tumor size increases [21, 22]. A recent systematic review estimates the 2-year local control rate of 69% for large (31–40 mm) BM treated with FSRT (30 Gy in five fractions) [22]. Surgery is of great significance for the patients with symptomatic BM larger than 3.0 cm [23]. With regard to postoperative stereotactic radiotherapy, a recent systematic review and meta-analysis suggested that the RN rate after SRS was low (6.9%) [24]. However, there is value in developing a safer irradiation method. Symptomatic BMs requiring resection are often large. High-dose radiation with postoperative SRS to a large cavity is associated with higher rates of RN. Eaton et al. analyzed the clinical outcome of 75 patients with a resection cavity > 3.0 cm who received SRS or FSRT, and concluded that fractionated radiosurgery is significantly associated with a decreased risk of RN for cavities 3–4 cm in size or larger [10]. In addition, because of the difficulty of postoperative cavity delineation, a 2 mm margin expansion for postoperative SRS was required, which is also associated with higher rates of RN [13, 25]. To overcome this shortcoming, preoperative SRS has been used. Patel et al. reported that preoperative SRS has reduced rates of symptomatic RN compared with postoperative SRS (2 years: 4.9% versus 16.4%, *P* = 0.010) [13]. In the current case series, 20 patients with BM who underwent neoadjuvant FSRT followed by resection were examined. Although this case series consisted of large tumors with a median maximum diameter of 37 mm and a median tumor volume of 17.6 cm^3^, no patients developed RN. This indicates the low radiotoxicity of preoperative FSRT for large brain tumors.

Preoperative fractionated SRT is a potential method of decreasing LMD recurrence. Suki et al. demonstrated that the risk of LMD was significantly higher with piecemeal tumor resection than with en bloc resection or SRS alone (SRS alone: hazard ratio for piecemeal, 5.8; *P* = 0.002, en bloc: hazard ratio for piecemeal, 2.7, *P* = 0.009) [12]. In clinical practice, the choice of piecemeal resection is often unavoidable when the tumor is large in size and/or is close to the eloquent areas. Few reports on optimal adjuvant radiation therapy for cases that underwent piecemeal resection have been published. Postoperative SRS, which has been recommended as an alternative to postoperative whole-brain irradiation [11], has the disadvantage of a high LMD rate (as high as 35%) [26]. Recently, Patel et al. reported that preoperative SRS had a reduced rate of LMD compared with postoperative SRS (2 years: 3.2% versus 16.6%, *P* = 0.010) [13]. Additionally, they reported that preoperative SRS had a rate of LMD similar to that of whole-brain irradiation (2 years: 3.5% versus 9.0%, *P* = 0.66) [2]. In this case series of 20 patients who underwent piecemeal resection, only one patient (5.0%) developed LMD. Preoperative FSRT has the potential to reduce intraoperative seeding of viable tumor cells beyond the operative field, resulting in a low risk of LMD.

Little has been revealed about the safety and diagnostic accuracy of preoperative FSRT. In this study, nausea occurred in one patient and simple partial seizures in two patients during radiation therapy. However, both symptoms were easily controlled by medication. Postoperatively, deterioration of paresis was occurred in two patients with tumors located near the central sulcus. The postoperative mortality rate within 30 days was 0%. We thus suggest the feasibility of preoperative FSRT. All cases could be accurately diagnosed as involving metastatic brain tumor. Although preoperative FSRT has the disadvantage of not achieving a histological diagnosis before treatment, it appears that an accurate diagnosis can be achieved after treatment.

This case series has some limitations. The analyzed data are from a single facility, there is a risk of patient selection bias, and a small sample size was evaluated. Furthermore, since the follow-up period was relatively short, it is necessary to perform careful follow-up in the future. Finally, with the FSRT method, there is a longer preoperative period than for SRS methods, so it is not suitable for patients requiring urgent surgery.

In conclusion, neoadjuvant FSRT appears to be a safe and effective approach for patients with BM requiring piecemeal resection. A multi-institutional prospective trial is needed to confirm this.

## Abbreviations

BM: Brain metastasis
CT: Computed tomography
CTCAE: Common terminology for adverse events
DR: Distant recurrence
FSRT: Fractionated stereotactic radiotherapy
LR: Local recurrence
LMD: Leptomeningeal disease
MRI: Magnetic resonance imaging
OS: Overall survival
RN: Radiation necrosis
SRS: Stereotactic radiosurgery
SRT: Stereotactic radiotherapy
